# Surface Properties of Aerated Ion-induced Whey Protein Gels

**DOI:** 10.1007/s11483-014-9384-x

**Published:** 2014-12-09

**Authors:** Marta Tomczyńska-Mleko, Konrad Terpiłowski, Stanisław Mleko, Cezary Kwiatkowski, Małgorzata Kawecka-Radomska

**Affiliations:** 1 Institute of Plant Genetics, Breeding and Biotechnology, University of Life Sciences in Lublin, Akademicka Street 15, 20-950 Lublin, Poland; 2 Department of Physical Chemistry-Interfacial Phenomena, Maria Curie Skłodowska University, M. Curie Skłodowska Sq. 3, 20-031 Lublin, Poland; 3 Department of Milk Technology and Hydrocolloids, University of Life Sciences, Skromna 8, 20-704 Lublin, Poland; 4 Department of Herbology and Plant Cultivation Techniques, University of Life Sciences, Akademicka 13, 20-950 Lublin, Poland; 5 Institute of Soil Science and Environmental Development, University of Life Sciences, Leszczyńskiego 7, 20-069 Lublin, Poland

**Keywords:** Whey protein, Surface properties, Gel, Roughness, Contact angle, Rheology

## Abstract

Aerated whey protein gels were formed using calcium chloride, magnesium chloride or iron (II) chloride induced gelation of pre-denatured protein dispersions. The structure of the obtained gel surface depends on the type and concentration of added salt. Higher cation concentration produced gels a with higher quadratic mean of the surface roughness and maximum roughness height. Aerated gels of optimal properties for retaining air bubbles were characterized by similar surface roughness. The surface topography is mainly responsible for changes in the wettability. The contact angle of the probe liquid sample depends on the liquid surface tension components. An approach based on the contact angle hysteresis (CAH) is suitable for determining the total value of the apparent surface free energy of such materials. An approach based on the components of apparent surface free energy (LWAB) only allows the calculation of the dispersion component and electron donor parameter of energy in the case of added magnesium and iron salt. Wettability, depending on the nature of the surface, can be described for the hydrophilic surface by the Wenzel model, and for the hydrophobic surface by the Cassie – Baxter model.

## Introduction

Whey proteins are becoming very popular as functional ingredients in foods. They enrich foods in the most valuable amino acids and shape their texture, mouthfeel, water and flavor holding capacity [1, 2]. Gelation is the most important functional property of whey protein and it is a key process to generate food texture [ 3]. Heating of whey protein solution above the denaturation temperature causes unfolding and aggregation of proteins. After cooling down, at low ionic strength, a thick solution can be obtained. Addition of ions will result in electrical shielding of charges and formation of gel. Previous papers introduced a novel method of obtaining whey protein aerated gels by simultaneous ion-induced gelation and aeration [4, 5]. Introducing a gas phase into a gel changes its texture, appearance, color and mouth-feel [1]. Aerated foods have a lower caloric value and aeration is a cost effective process by increasing the product volume. Application of gels can be extended by using the aeration process. More or less sophisticated methods could be used to obtain different three-dimensional, microstructured aerated gels. For whey protein gels the gel capacity to retain air bubbles depends on pH, protein concentration and concentration of induced ion [5]. Pulsed laser submicron foam formation has been demonstrated in gelatine [6]. Whey protein gel as a natural and easily biodegradable product could be used as a matrix for of active ingredients release and tissue engineering [7]. Aeration of the gel could create a very new product of different susceptibility to dissolve in the human stomach and ability to float.

Control of surface roughness, pore size and shape as well as surface wettability in polymers and biopolymers is very important in material science, medicine, biotechnology and food science.

The use of a polarizing microscope allows better illumination of the sample by the waves of the same direction and the same length. There is no known surface analysis of whey proteins using a polarizing optical microscope except the paper by of Tzoumaki et al. [8]. They used the method of optical polarization microscopy to obtain images of the surface of mixed chitin/whey protein gels. Using this method it was possible to distinguish the areas where the gelled whey protein occurred. Szcześ et al. [9] used polarization microscopy to observe crystallization under the influence of a magnetic field. They investigated morphology changes taking place during the melting and crystallization of freshly precipitated calcium phosphate.

Microscopic methods allow viewing of of the gel surface with large magnification, however, they do not allow quantification of surface roughness. Optical profilometer is an instrument allowing surface roughness investigation. There are no studies on the gel surface using optical profilometry. Generally the study on the gel surface is scarce. Chen [10] presented an interesting review of “surface texture” of food products in which it is regarded as all surface features, obtained from the visual sensations, touch and feel in the mouth. Nayebzadeh et al. [11] observed natural, not-dried gels using a modified, steam filled column method of scanning electron microscopy. The computer program based on microscopic images calculated the parameters determining the surface properties of mixed whey protein/xanthan gels. Similarly, using a computer image analysis roughness of whey protein gels using a laser scanning confocal microscopy was evaluated [12, 13].

Another important surface property is its wettability. In the approach of van Oss et al. [14] the surface free energy is expressed as the sum of two constituents: apolar Lifshitz-Van der Waals (*γ*_*i*_^*LW*^) and Lewis acid–base (*γ*_*i*_^*AB*^):

1 $$ {\gamma}_i={\gamma}_i^{LW}+{\gamma}_i^{AB} $$

Besides dispersion interactions the component (*γ*_*i*_^*LW*^) includes the dipole orientation and the induction ones which were considered to be polar earlier. According to Good et al. [15] the component of acid–base interactions (*γ*_*i*_^*AB*^) can be expressed by the geometric mean:

2 $$ {\gamma}_i^{AB}=2{\left({\gamma}_i^{+}{\gamma}_i^{-}\right)}^{1/2} $$

Based on such model the adhesion work can be written by means of the constituents:

3 $$ {W}_A={\gamma}_l\left(1+ \cos \theta \right)=2\sqrt{\gamma_s^{LW}{\gamma}_l^{LW}}+2\sqrt{\gamma_s^{+}{\gamma}_l^{-}}+2\sqrt{\gamma_s^{-}{\gamma}_l^{+}} $$

The quantity of the constituent *γ*_*s*_^*LW*^ and the parameters *γ*_*s*_^+^, *γ*_*s*_^−^ of surface free energy can be determined from Eq. 3 measuring the wetting angle of three different liquids of known values of surface tension constituents: *γ*_*l*_^*LW*^, *γ*_*l*_^+^ and *γ*_*l*_^−^. Then there should solved a set of three equations with three unknown values (*γ*_*s*_^*LW*^, *γ*_*s*_^+^, *γ*_*s*_^−^) which allows to determine the energy components (*γ* ^*LW*^, *γ* ^*AB*^ and finally its total value.

Selection of liquids used for measuring wetting angles by van Oss et al. [14] and van Oss et al. [16] is essential in determination of surface free energy of solids. The most suitable for calculation of components of surface free energy there proved to be a set of three liquids of which one is apolar of high surface tension and the other two are polar liquids differing significantly in the quantities *γ*_*l*_^*LW*^, *γ*_*l*_^+^ and *γ*_*l*_^−^. In practice there is usually used diiodomethane (*γ*_*l*_ = 50, 8 *mJ*/*m* ^2^) or 1-bromonaphtalane (*γ*_*l*_ = 44.4 *mJ*/*m* ^2^) and water and formamide as polar liquids [17, 18].

The above mentioned approach gives good results on highly energetic surfaces that is on which liquids have low wetting angles (e.g. metals). However, using this approach for calculation of energy on hydrophobic surfaces leads to negative values of elements of one of polar constituents, usually the electron – acceptor one. Such a result is pointless from the physicochemical point of view and then the surface free energy is limited only to one dispersion component.

The method for calculation of solid surface free energy from hysteresis was proposed by Chibowski [19, 20]. It is based on hysteresis of wetting angle and three experimentally measured parameters: ascending and receding wetting angles as well as surface tension of liquid. The value of surface free energy is also significantly affected by the presence of liquid film formed during receding of liquid front or due to adsorbance of liquid vapours or droplet spreading on the solid surface.

4 $$ {\gamma}_s=\frac{\gamma_l{\left(1+ \cos {\theta}_a\right)}^2}{2+ \cos {\theta}_r+ \cos {\theta}_a} $$

where: γ_s_ – apparent surface free energy, γ_l_ – liquid surface tension, θ_a_ – advancing contact angle, θ_r_ – receding contact angle

Surface free energy calculated from hysteresis of wetting angle is somehow dependent on physicochemical properties of the liquid. This allows to use this approach for comparison of different surfaces. To avoid depending only on physicochemical properties of the liquid, it is possible to calculate the average energy obtained from measuring hysteresis of wetting angle of different liquids e.g. water, formamide or ethylene glycol. While applying such liquids as diiodomethane, hexadecane or hydrocarbons it is possible to determinate dispersive component of apparent surface free energy with a small error from Eq. 4. Contact angle measurements were found helpful in explaining structure of gels and films. Białopiotrowicz [21] concluded that starch gel surface tries to maintain maximal hydrophobic character with polar domains created by the functional glucose groups with the branched chain of amylopectin directed into air. For gelatin films a monopole-donor character caused by the existence of carbonyl or ionized carboxyl groups was ascertained [22]. There is no research on wettability of whey protein gels. Research on surface properties of whey protein aerated gels can be important because the surface shape, roughness and porosity are essential for such processes such as enzymes hydrolysis, reactivity of the surface in contact with chemical reagents, adhesion and diffusion of the active ingredients, deposition of micro-organisms on the surface of the product. Measurements of contact angle on the surface of a gel is a difficult issue. According to some scientists, measurements of contact angles require the solid surfaces to be rigid, smooth and homogeneous, so the Young’s equation is the appropriate equilibrium condition. The solid surfaces should be as inert as possible so that effects, such as swelling and chemical reactions are minimized [23]. Preliminary research showed that the aerated whey protein gels are rigid and the surface prior to analysis was dry. At the time of measurements no changes in contact angle values were observed. Some controversy is also connected with measurements of contact angles on rough surfaces. As yet there are no general guidelines regarding how smooth a solid surface must be for surface roughness not to have impact on the contact angle measurements [24]. Nevertheless, it has been mathematically proven that the Wenzel equation yields the apparent contact angle on sawtooth surfaces when the size of the drop is very large compared with the scale of the roughness [25].

In the present study, we used different methods to characterize surface properties of the aerated whey protein gels - a new product which can be used for an active ingredient release in human organism.

## Materials and Methods

Whey Protein Isolate (WPI) (88.0 % protein) was purchased from Arla Foods Ingredients (Viby, Denmark). The protein content was determined by analyzing nitrogen using the macro Kjeldahl method and calculating protein as N × 6.38. The mineral composition was determined by an atomic absorption spectrometry using a Varian Spectra 280 FS (Varian, Inc., Palo Alto, USA). The result is the mean of three replications.

No gelation was observed at heating of 8 % *w/w* protein solution for 30 min at 80 °C. It was caused by a low content of minerals in the investigated WPI (Table 1). Preliminary research allowed choosing conditions of protein concentration, salt concentration and pH to obtain gels with the best texture and capability to hold air bubbles (Table 2). The WPI dispersions were made by hydrating in distilled water at 22 °C for 30 min using a magnetic stirrer. The pH of the native protein solution was 6.68. For some samples the pH of the dispersions was adjusted to 7.34 (average value between 6.68 and 8). Dispersions were heated in water bath for 30 min at 80 °C and after heating immediately cooled down. Calcium chloride, magnesium chloride or iron (II) chloride was added in the concentrations 10, 20 or 30 mM (see Table 2). Immediately after adding the salt, solutions were foamed for 30 s at 2000 rpm using Compact Digital Lab Mixer (Cole-Parmer, Montreal, Canada). The aerated gels were stored for 20 h at 7 °C, equilibrated at 21 °C for 2 h and subjected to evaluation of their rheological, structural and surface properties.

**Table 1:** Mineral composition of whey protein isolate

Element	Concentration % (*w/w*) or ppm
Na	0.54 %
K	1.34 %
Ca	0.05 %
Mg	0.03 %
P	0.24 %
Cl	0.05 %
Cu	2.20 ppm
Fe	17.0 ppm
Pb	0.29 ppm
Cd	0.05 ppm
As	0.02 ppm

**Table 2:** Types of investigated aerated whey protein gels

Salt type	Prot. conc.% (*w/w*)	Salt conc. mM	pH	Air content (*v/v*)	Average bubble size μm
MgCl_2_	7.0	20; 30	7.34	40.9; 47.7	63; 286
CaCl_2_	8.0	20; 30	6.68	37.6; 45.6	77; 393
FeCl_2_	7.5	10; 30	6.68	47.5; 52.8	57; 162

### Air Fraction Measurements

The density of aerated and non-aerated gels was determined by the flotation method described by Zuniga and Aguilera (2009). Gel samples were cut into the form of cubes with the edges of about 6 mm and were placed in a measuring flask with a capacity of 50 cm^3^. The flask was filled with distilled water. Gels, flasks with the gel and water, and flasks with water were weighed.

The density of aerated and non-aerated gels was calculated from the formula:

$$ \uprho \mathrm{G}=\frac{\uprho w\ast mG}{mG+mcw- mcwG} $$

where:

ρG: density of the non-aerated gel in g/cm^3^ (aerated - ρAG)
ρ_W_: density of water at 25 °C (0,99705 g/cm^3^)
mG: mass of the gel (g)
mcw: mass of the flask with water (g)
mcwG: mass of the flask with the gel and water (g).

Fraction of air (ϕ) has been calculated using the formula:

$$ \upphi =\frac{1-\rho AG}{\rho G}100\% $$

All measurements represent the arithmetic means of three replicates.

### Bubble Size Measurements Using Turbiscan

After aeration the gels were poured into a flat-bottomed glass cylindrical sample cell. The average bubble size in the gels was investigated by the Turbiscan apparatus (Formulaction, L’Union, France). The samples were scanned by a pulsed near infrared light source (wavelength 880 nm) and two synchronized detectors were used to collect the transmitted and backscattering lights. The obtained data was expressed as a percentage intensity of the transmission or backscattering. The average size of the air bubbles dispersed in the gel was calculated using the Turbiscan Lab expert software (Formulaction, L’Union, France). All measurements were made in triplicate and the results present the arithmetic means.

### Dynamic Rheology

Small strain frequency sweeps rheological measurements were performed using the RS300 rheometer (ThermoHaake, Karlsruhe, Germany). Serrated parallel steel plate geometry (35 mm diameter, gap size 2 mm) was used to limit the potentiality of sliding effects.

### Surface Roughness

After aeration the pre-formed gels were poured into Petri dishes and the aerated gel surface was observed using an optical profilometer GT Contour Surface Metrology (Veeco, Tucson, USA). Surface roughness was determined using Vision64 (Veeco, Tucon, USA).

### Contact Angles Measurements

Advancing and receding contact angles of the probe liquids on the gel surfaces were measured using the contact angle meter GBX (France) equipped with a temperature and humidity controlled measuring chamber and digital camera. The measurements were conducted at 20 °C and 50 % relative humidity. A 6 μL droplet from a syringe was gently settled on the sample surface with help of an automatic deposition system. The advancing contact angle was evaluated from the droplet shape by the computer program Win Drop++. Then 2 μL of the droplet volume were sucked into the syringe and the receding contact angle was calculated by the mentioned program. The advancing and receding contact angles were measured for up to 10 droplets of each probe liquid.

### Microstructure

Microstructure of the aerated gels was viewed using the scanning electron microscopy and the polarized light microscopy. The samples of the aerated gels were fixed by immersion in 2.5 % glutaraldehyde solution in 0.1 M sodium cacodylate buffer. The samples were dehydrated in serial dilutions of ethanol and acetone and dried at the critical point in liquid carbon dioxide. The preparations were coated with gold using a vacuum evaporator EMITECH K550x (Emitech, Ashford, United Kingdom). They were viewed and photographed using a scanning electron microscope VEGA II LMU (Tescan, Canberra, USA).

After aeration the pre-formed gels were poured into Petri dishes and the aerated gel surface microstructure was observed using a polarizing optical microscope Eclipse E600 Pol (Nikon, Tokyo, Japan).

## Results and Discussion

### Air Incorporation in the Gels

The air content in the investigated gels was from 37.6 to 52.8 % (*v/v*) (Table 2). The increased salt concentration caused an increase in air content. It was probably the effect of higher aggregation of protein at higher ion concentration. Large aggregates form coarser structure capable of holding larger bubbles. The average bubble size increased for about 3–4 times at higher ion concentration (Table 2).

### Microstructure

**Fig. 1:**
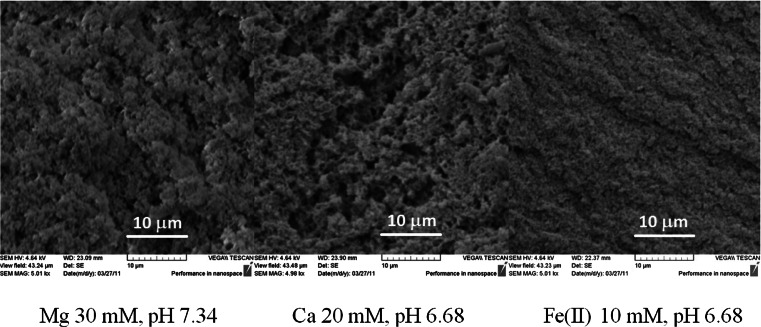
SEM images of the gel surfaces

Figure 1 shows SEM images of the gels obtained by ion-induced gelation process. Different cations, different salt concentration and different pH resulted in differences in protein aggregation. Different size of protein aggregates influences roughness of the gel surface. The interesting fact is that despite differences in cation type, its concentration and pH value of dispersion, the obtained gel structures were the most optimal for retaining air bubbles. This was observed sensorially in the preliminary research. Too weak gels were not able to hold air bubbles and they moved to the gel surface and too strong gels did not form homogeneous structure after storing for 20 h at 7 °C. The gel structure formed “in statu nascendi” after adding ions was destroyed at the foaming process, and broken strong gel could not reverse its microstructure. It is difficult to find an objective method to measure which conditions are the best to maintain air bubbles (e.g. air fraction or aerated gel stability), as strong gel also was “foamed” holding air between broken microgels. Such a material composed of broken microgels could not be used as a matrix for active ingredients release as it does not form three-dimensional structure in one piece. The best method to optimize conditions for producing aerated gels was to measure storage modulus value and tangent delta. Three-dimensional structures of the gels capable of holding air bubbles were the most elastic and were characterized by the highest storage modulus and the lowest tangent delta values. Broken, not rehealed gel and too weak gel have lower storage modulus value and higher tangent delta. Based on this preliminary research, two different salt concentrations were chosen for the same cation (Table 2). The highest value of storage modulus and the lowest value of loss tangent were observed for the gels with the addition of 30 mM Mg^2+^, 20 mM Ca^2+^ and 10 mM Fe^2+^ (Table 3). These gels could be used as matrices for active ingredient release as the texture of such gels should be as solid as possible. It is interesting to note that these gels were characterized by different air content and different average bubble size (Table 2). It means that the rheological properties of aerated gels are mostly determined by the gel matrix and not the air content and average bubble size.

**Table 3:** Surface roughness parameters and small-strain rheological data for whey protein aerated gels: quadratic mean of the surface roughness (R_q)_, maximum roughness height (R_t)_, storage modulus (G’), tangent of the phase angle (tanδ

Aerated gel	R_a_ (nm)	R_q_ (nm)	R_t_ (μm)	G’ (Pa)	tanδ
Mg 20 mM	222 ± 40	364 ± 56	1.8 ± 0.3	1568 ± 23	0.134 ± 0.002
Mg 30 mM	536 ± 95	853 ± 61	4.5 ± 0.5	2096 ± 31	0.128 ± 0.004
Ca 20 mM	704 ± 74	806 ± 71	4.6 ± 0.7	6246 ± 49	0.136 ± 0.005
Ca 30 mM	1270 ± 30	993 ± 81	6.2 ± 0.7	4221 ± 42	0.143 ± 0.002
Fe (II) 10 mM	483 ± 49	651 ± 53	3.5 ± 1.2	2520 ± 19	0.104 ± 0.001
Fe (II) 30 mM	1393 ± 336	1840 ± 38	15.7 ± 2.4	1982 ± 27	0.112 ± 0.003

### Surface Roughness

**Fig. 2:**
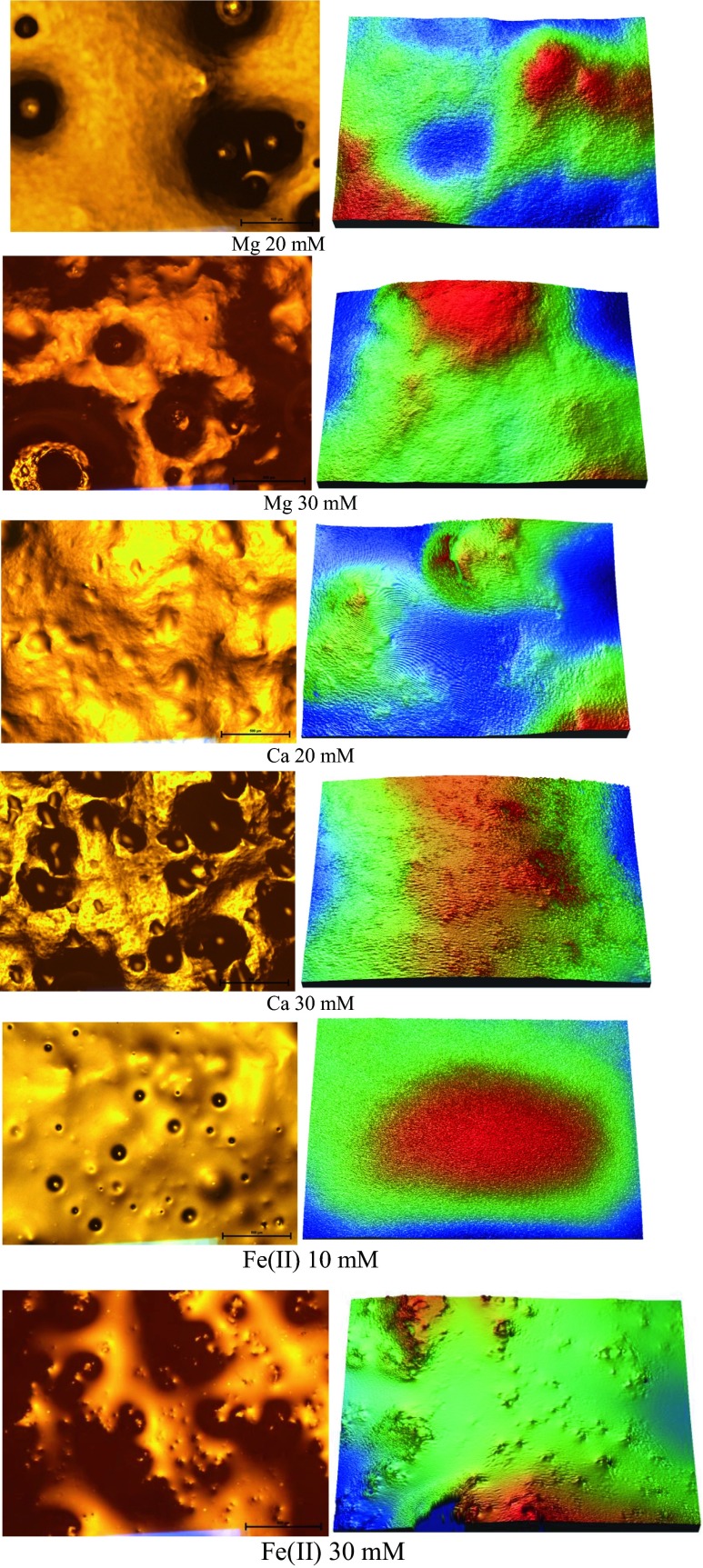
Polarized light microscope (*left*) and surface optical profilometer images of the aerated gels, microscope image bar = 500 μm, profilometer image size = 156 × 117 μm

**Fig. 3:**
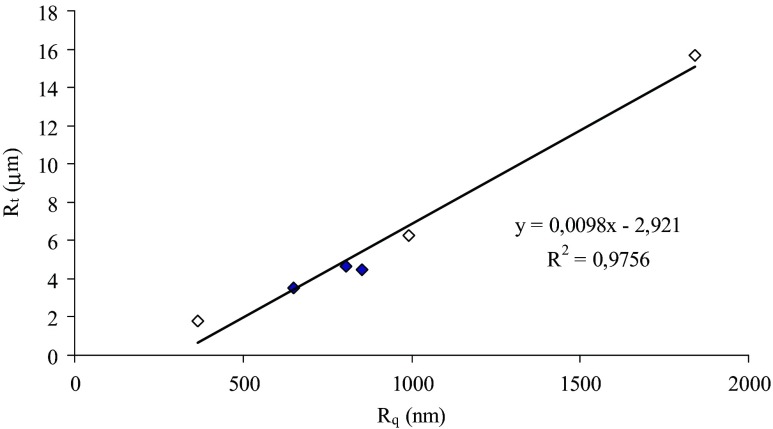
Correlation between the quadratic mean of the surface roughness (R_q)_ and the maximum roughness hight (R_t)_ for the aerated gels (full diamonds - aerated gels with higher storage modulus value for the same cation type)

Figure 2 shows the images of the surface of aerated gels obtained using a polarized light microscope and a surface optical profilometer. Higher ions concentration resulted in rougher structure and it can be easily seen on the polarized microscope images. This is not so obvious for the surface optical profilometer images, but calculation of surface roughness parameters reveals that higher concentration of cations produced gels with a higher quadratic mean of the surface roughness and maximum roughness height (Table 3). Chen et al. [13] observed that whey protein gel without the salt addition had a very smooth surface with R_q_ and R_a_ of 0.20 and 0.18 μm, respectively, but the gel containing 200 mM NaCl had a much rougher surface with large R_q_ and R_a_ (2.39 and 1.91 μm, respectively). This is probably due to the increased protein aggregation caused by high concentrations of salt [26]. Nayebzadeh et al. [11] noticed that xanthan has a surface smoothing effect on the heat-set whey protein gels. Xanthan molecules appeared to spread out uniformly at the surface, filling the holes and void spaces within the protein network. There is a linear correlation between the quadratic mean of the surface roughness and the maximum roughness height (R^2^ = 0.98) (Fig. 3). In addition, it was observed that the points corresponding to the aerated gels with optimal properties for retaining air bubbles were characterized by similar surface roughness, which suggests that the most favorable conditions for the creation of aerated gels occur at optimal protein aggregation. It seems that optimal aggregation for creating aerated gels depends on cation type, even when cations have the same valence. This study suggests that higher protein aggregation on a microstructural level of micrometers results in higher roughness on the surface of the gel.

### Contact Angles of Probe Liquids and Apparent Surface free Energy

As can be seen in Table 4, the increase in the CaCl_2_ concentration does not affect significantly the change of contact angles of tested liquids. For the aerated gels obtained at 20 mM CaCl_2_ the water advancing contact angle is 121.0° ± 14.1 but for the sample with the higher CaCl_2_ concentration it increases slightly to the value 128.8° ± 7.0. Much greater increase is found for the wetting angles measured for formamide. For the sample with lower concentration of CaCl_2_ it is 32.9° ± 3.1, whereas for the sample with the higher concentration of salt it is 92.4° ± 4.2. There are only small changes of the contact angle of diiodomethane, which is an apolar liquid and “reacts” with the surface only in the dispersive way.

**Table 4:** Advancing and receding contact angles [deg.] of probe liquids measured for glass plates covered by protein aerated gel with different concentration of CaCl_2_ and apparent surface free energy [mJ/m^2^] calculated from CAH and LWAB approaches

Liquid	θ_a_	θ_r_	γ^tot^_s (CAH)_	γ^LW^_s_
CaCl_2_ 20 mM				
water	121.0 ± 14.1	96.6 ± 10.2		
formamide	32.9 ± 3.0	23.6 ± 5.4	30.7 ± 2.6	24.0 ± 1.4
diodomethane	67.9 ± 3.2	47.5 ± 2.8		
CaCl_2_ 30 mM				
water	128.8 ± 7.0	117.9 ± 5.8		
formamide	92.4 ± 4.0	76.2 ± 2.4	25.0 ± 2.3	27.8 ± 0.7
diodomethane	61.3 ± 1.4	47.1 ± 3.1		

The increase in MgCl_2_ concentration affects significantly the increase of surface hydrophobic properties (Table 5). The advancing water contact angle increases from 63.0° ± 2.3 up to 114.2° ± 5.9. A similar increase can be observed for the contact angles of formamide. The formamide contact angles increase by about 32°. It is worth noting that the contact angles of diiodomethane decrease from 64.7° ± 3.9 for the surface covered with gel with the addition of 20 mM to 39.4° ± 7.4 for the surface with 30 mM MgCl_2_ aerated gel.

**Table 5:** Advancing and receding contact angles [deg.] of probe liquids measured for glass plates covered by protein aerated gel with different concentration of MgCl_2_ and apparent surface free energy [mJ/m^2^] calculated from CAH and LWAB approaches

Liquid	θ_a_	θ_r_	γ^tot^_s (CAH)_	γ^LW^_s_	γ^−^_s_
MgCl_2_ 20 mM					
water	63.0 ± 2.3	23.8 ± 3.2	38.7 ± 1.3	26.5 ± 1.2	43.4 ± 0.1
formamide	74.8 ± 3.4	68.6 ± 5.2			
diodomethane	64.7 ± 3.9	49.2 ± 3.4			
MgCl_2_ 30 mM					
water	114.2 ± 5.9	98.2 ± 6.8	26.6 ± 0.4	39.8 ± 3.0	3.5 ± 0.9
formamide	106.8 ± 6.3	98.8 ± 5.8			
diodomethane	39.4 ± 7.4	28.3 ± 6.7			

Table 6 presents the change of probe liquids contact angles measured on the glass plates covered with aerated gel with the addition of 10 and 30 mM FeCl_2_. Contrary to the earlier described surfaces, the increase in the salt amount causes the decrease in the contact angles of the tested polar liquids i.e. water and formamide. The value of water contact angle drops from 91.0° ± 8.2 for the surface with a smaller FeCl_2_ concentration to 76.9° ± 4.5 when the FeCl_2_ concentration is 30 mM. Taking into account changes of surface free energy (Table 4) on the surface of aerated gel with the addition of calcium salt along with the increasing surface hydrophobic properties, drop in the value of surface free energy is observed. If the apparent surface free energy for these surfaces was calculated based on the contact angle hysteresis approach [19], then with the increasing concentration of CaCl_2_ its value decreased from 30.7 ± 2.6 mJ/m^2^ to 25.2 ± 2.3 mJ/m^2^. Applying the LWAB approach [14], due to the surface hydrophobic character (Table 4), the total value of apparent surface free energy was confined only to the dispersion component which increases insignificantly from 24.02 ± 1.4 mJ/m^2^ to 27.8 ± 2.3 mJ/m^2^ for the surface with a higher concentration of CaCl_2_. Taking into account the topography of the considered surface (Fig. 2), the increase in calcium chloride concentration results in the increasing surface roughness. Thus associating it with the surface hydrophobic character (Table 4), it should be stated that wettability of these surfaces is described by the Cassie – Baxter model [27].

**Table 6:** Advancing and receding contact angles [deg.] of probe liquids measured for glass plates covered by protein aerated gel with different concentration of FeCl_2_ and apparent surface free energy [mJ/m^2^] calculated from CAH and LWAB approaches

Liquid	θ_a_	θ_r_	γ^tot^_s (CAH)_	γ^LW^_s_	γ^−^_s_
FeCl_2_ 10 mM					
water	91.0 ± 8.2	72.8 ± 4.2	35.1 ± 1.4	37.1 ± 3.1	5.6 ± 2.8
formamide	75.3 ± 6.1	69.5 ± 7.5			
diodomethane	41.5 ± 2.0	32.2 ± 2.1			
FeCl_2_ 30 mM					
water	76.9 ± 4.5	57.1 ± 6.1	39.6 ± 0.9	36.0 ± 3.5	19.9 ± 1.0
formamide	74.2 ± 8.8	72.4 ± 7.8			
diodomethane	43.6 ± 6.1	30.9 ± 2.6			

**Fig. 4:**
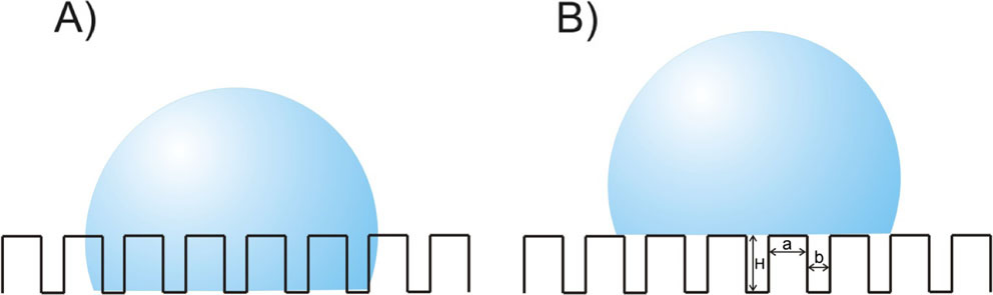
Schematic picture of a typical rough surface **a** droplet in the Wenzel state, **b** droplet in the Cassie-Baxter state

There are two basic types of rough surface wettability by the liquid. If a drop of the liquid wets the surface completely filling all cavities (Fig. 4a), the wetting is compatible with the Wenzel model [28]. In contrast (Fig. 4b), when the surface is hydrophobic and a drop of liquid (water) does not wet the surface completely and is only supported by the peaks of the roughness, the Cassie – Baxter model of wettability is valid. In the second model higher values of contact angles are obtained [27].

Describing changes in the apparent surface free energy on the surface of glass plates covered with hydrogel with the magnesium salt addition [Table 5], there is observed drop of surface energy with the increasing concentration of MgCl_2_ in the surface layer. With the CAH approach, the energy dropped from 38.7 ± 1.3 mJ/m^2^ to 26.6 ± 0.4 mJ/m^2^ for the surface where the MgCl_2_ concentration was 30 mM. Similar to the case of the surface with the calcium salt addition using the LWAB approach, it was not possible to calculate the total value of apparent surface free energy. As can be seen in Tab. 4 the value of the dispersion component increases with the increasing magnesium salt concentration by about 13.3 mJ/m^2^. However, there is observed a significant decrease of the value of electron – donor component on the surface with 30 mM MgCl_2_ and it is lower by about 40 mJ/m^2^. Comparing the data from Table 3, it is possible to draw the conclusion that similar to the case of hydrogels with calcium chloride, the differences in the value of apparent surface free energy are caused by changes in the surface topography. The average surface roughness increases 2.5 times. Comparing these values with the measured contact angles of the tested liquids on these surfaces (Table 5) due to the increase in magnesium salt concentration, the Wenzel model [28] changes into the above mentioned Cassie – Baxter model, that is the surface character changes from the hydrophilic to the hydrophobic one.

As can be seen in Table 6 in the case of glass plates covered with hydrogels with the addition of iron salt, the total value of surface free energy increases with the increasing amount of salt. The apparent surface free energy increases from 35.1 ± 1.4 mJ/m^2^ to 39.6 ± 0.9 mJ/m^2^ for the surface with the addition of 30 mM FeCl_2_. The dispersion component of surface energy free energy remains practically without any changes. However, there is the increase of electron – donor component value, when it was only 5.6 ± 2.8 mJ/m^2^ for the surface where the FeCl_2_ concentration was 10 mM, to 19.9 ± 1.0 mJ/m^2^ for the surface with the higher concentration of salt. Analysing the data in Table 3 about surface roughness, it is found that with the higher addition of FeCl_2_ roughness is described with the micro scale as evidenced by a significant value of deflection in the case of R_a_ and a high value of R_t_ which is as much as 15.7 ± 2.4 μm. Thus the increase in the value of surface free energy can be explained by the increase of the surface roughness in the micro scale, that is wettability from the Wenzel model, and the existence of a number of polar functional groups on the surface originating from FeCl_2_.

## Conclusions

The structure of the obtained surfaces depends on the type and concentration of the added salt. Higher cation concentration produced gels with a higher quadratic mean of the surface roughness and maximum roughness height. The aerated gels with optimal properties for retaining air bubbles were characterized by similar surface roughness, which suggests that the most favorable conditions for the creation of aerated gels occur at optimal protein aggregation. The surface topography is mainly responsible for changes in the wettability: micro- and nano-roughness, or both occurring simultaneously. The contact angles of the probe liquid sample depend on the liquid surface tension components. An approach based on the contact angle hysteresis (CAH) is suitable for determining the total value of the apparent surface free energy of such materials. An approach based on the components of apparent surface free energy (LWAB) allows the calculation of the dispersion component and electron donor parameter of energy in the case of added magnesium and iron salt. The wettability, depending on the nature of the surface, can be described for the hydrophilic surface by the Wenzel model, and for hydrophobic surface by the Cassie – Baxter model.
